# Link Clustering with Extended Link Similarity and EQ Evaluation Division

**DOI:** 10.1371/journal.pone.0066005

**Published:** 2013-06-19

**Authors:** Lan Huang, Guishen Wang, Yan Wang, Enrico Blanzieri, Chao Su

**Affiliations:** 1 College of Computer Science and Technology, Jilin University, Changchun, China; 2 Department of Information and Communication Technology, University of Trento, Povo, Italy; Cinvestav-Merida, Mexico

## Abstract

*Link Clustering* (LC) is a relatively new method for detecting overlapping communities in networks. The basic principle of LC is to derive a transform matrix whose elements are composed of the link similarity of neighbor links based on the Jaccard distance calculation; then it applies hierarchical clustering to the transform matrix and uses a measure of partition density on the resulting dendrogram to determine the cut level for best community detection. However, the original link clustering method does not consider the link similarity of non-neighbor links, and the partition density tends to divide the communities into many small communities. In this paper, an *Extended Link Clustering* method (ELC) for overlapping community detection is proposed. The improved method employs a new link similarity, *Extended Link Similarity* (ELS), to produce a denser transform matrix, and uses the maximum value of EQ (an extended measure of quality of modularity) as a means to optimally cut the dendrogram for better partitioning of the original network space. Since ELS uses more link information, the resulting transform matrix provides a superior basis for clustering and analysis. Further, using the EQ value to find the best level for the hierarchical clustering dendrogram division, we obtain communities that are more sensible and reasonable than the ones obtained by the partition density evaluation. Experimentation on five real-world networks and artificially-generated networks shows that the ELC method achieves higher EQ and *In-group Proportion* (IGP) values. Additionally, communities are more realistic than those generated by either of the original LC method or the classical CPM method.

## Introduction

The need for community structure detection originates from the study of complex networks [1], [2], and aims to identify a system of sub-networks (or communities), whose nodes are tightly linked via the original network topology. The network has a community structure when nodes within the same community have more links than nodes belonging to different communities. The community structure exists as a property of the topology in many real complex networks, and such structures have been reported in social networks such as acquaintance networks [1]–[4] and collaboration networks [2], [5], [6], technological networks (word associations [7], [8], World-Wide Web [4], [6], air transportation [6]), and biological networks (protein-protein interaction [5], [9], metabolic networks [5], [8], [10]–[12]). When the community structure of a network is already known, it can be easily represented as an attribute of the nodes, as in the case of artificially-generated networks [1], [4], [10]. This is also true for some real-world networks used as testing benchmarks; for example Zachary’s karate club network [1]–[4] and US college football network [1]–[4]. Otherwise, in order to identify the community structure, it is necessary to analyze the relationship between the topology of possible communities and the overall topology of the network. When more than one community exists, the community structure can be disjoint (communities which have no nodes in common) such as in a social network representing exclusive social groupings by interest or background [1]–[4], hierarchical (one community includes the other) such as the hierarchical organization of modularity in metabolic networks [11], or overlapped (two communities may have some nodes in common) such as a large fraction of proteins belonging to several protein complexes simultaneously [12].

In 2002, the study of community structure detection in social and biological networks was initiated by Girvan and Newman [1]. In that paper, the authors describe community structure as a property of the topology, and provide a hierarchical clustering method based on link “betweenness” scores, as a means of identifying it. By 2004, Newman proposed a hierarchical clustering method based on greedy techniques [2]. Further advances in this particular area of research have resulted in additional methods for community detection and complex metabolic networks analysis, such as fuzzy c-means clustering [3], fitness function local optimization algorithms [4], and simulated annealing algorithms [10] to name a few.

Initially, the first methods proposed for community structure detection, restricted a node to being only a member of one community and thus simplified the overall structure of the communities to be found. This restriction allowed these methods to gain some computational advantages; however, for real networks such as social networks [5], [6], technological networks [6]–[8] and biological networks [11], [12], a node may genuinely belong to different communities simultaneously, and sometimes, many nodes in the network cannot be divided into separate communities without loss of generality. Hence, traditional methods are inadequate in identifying appropriate communities when overlaps are significant [5].

The phenomenon of community overlap was first investigated by Palla and his co-workers *et al.* in 2005 [5], where they proposed the *Clique Percolation Method* (CPM), which was subsequently widely used for overlapping community structure detection. Thereafter, Zhang *et al*. used the c-means clustering method for overlapping communities detection [3]. Another method by Breve *et al*., utilized the concept of population competition by random-deterministic walk to visit neighbor nodes as a means of detecting overlapping communities in complex networks [13]. Lancichinetti *et al*. presented an order statistics local optimization method based on the local optimization of a fitness function that expresses statistical significance of clusters with respect to random fluctuations. The advantage of this method is that it is able to handle different types of datasets as well as the subtleties of community structure for detecting overlapping communities, hierarchies and community dynamics [6]. In 2012, Zhang *et al*. developed a new regularized sparse random graph model [9] that combines the smooth regularizer and the objective function of the sparse random graph model. This method provides the capability to analyze overlapping of the various structural functional units in *Protein-protein Interaction* (PPI) networks.

The traditional agglomerative and hierarchical algorithms, such as Newman algorithms [1], [2], build hierarchical clustering trees on nodes, and most of the methods for detection of overlapping communities, like the above mentioned CPM method [5] and the c-means clustering method [3], focus directly on grouping nodes as well. It was Evans and Lambiotte [7] who were the first to propose clustering links instead of nodes, using the line graph of an undirected graph for overlapping communities detection. In a different approach, Ahn *et al.* [8] chose the Jaccard index of the neighborhoods of two nodes for analyzing links, called *Link Clustering* (LC), and successfully proved its viability, which brought about a whole new perspective for overlapping community study in 2010. Subsequently, Kalinka published the R language package “linkcomm” which is based on LC and oriented to social network clustering [14].

Research activity related to community detection is also comprised of activities related to the development of measures to evaluate the community structure itself. Newman first defined the quality function “modularity” Q to measure whether a community structure is meaningful [2]. A high value of Q represents a good community structure, and if a community structure has no more within-community edges than would be expected by random chance, Q will be 0. Although modularity can be used for evaluating the results of community detection, it cannot be applied directly to the evaluation of overlapping communities. Kapp *et al*. [15] proposed *In-group Proportion* (IGP), a measure of cluster quality based on the idea of prediction accuracy, and is able to measure community structures that are either overlapping or not. As an extension of modularity, Shen *et al*. proposed EQ to address Q’s limitations [16]. Recently, Ahn *et al*. used partition density for evaluating the detection of overlapping communities in the link clustering methodology [8].

In this paper, we propose an *Extended Link Clustering* (ELC) method which is based on Ahn’s link clustering [8] and Shen’s EQ evaluation [16]. In fact, we observe that the original link clustering method does not consider the link similarity of non-neighbor links, and the determination of the level where to cut the dendrogram based on partition density tends to divide the network into many small communities. The improved method employs an *Extended Link Similarity* (ELS) to get a denser transform matrix, and uses the maximum EQ value as a means of determining the optimal cut level of the dendrogram. The ELS transform considers the neighbor and non-neighbor links at the same time, and enhances the transform matrix’s capability for clustering and analysis. Meanwhile, using the EQ value instead of partition density to cut the dendrogram may define communities that are more sensible and reasonable than the ones obtained by the original method.

ELC is empirically evaluated against state-of-the-art methods. In the experiments on five real-world networks, such as Karate network [17], Dolphin network [18], US politics network [19] and Football network [1], Y2H (yeast two-hybrid) network [20] and artificially-generated networks, ELC achieves more reasonable partition results in the original network space than the original link clustering method and the classical CPM method. In most cases, it also reaches higher EQ and IGP values of overlapping. Overall, the final communities are more sensible and reasonable when compared to real world phenomena. Experiments on artificially-generated networks allowed for the study of the behavior of the three methods under different conditions for average degree and proportion 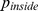. The results on the real-world datasets are compatible with the analysis done on the artificially-generated networks. Overall our study suggests that ELC should be used with low average degree when a rather high value of proportion 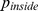 is expected.

## Materials and Methods

### Data Source

To evaluate the viability of ELC and to be able to compare its performance against other methods, we selected five real-world networks and a range of artificially-generated networks. The five real-world datasets contain some of the most relevant networks used by the research community, such as the Karate network [17], Dolphin network [18], US politics network [19], Football network [1] and Y2H (yeast two-hybrid) [20]. The artificially-generated networks are built using a random procedure based on known modular structures similar to that found in Newman’s [1] and Guimera’s papers [10].

1. Karate network (Zachary's Karate Club) [17] is a social network of friendships between 34 members of a karate club at a US university. It is among the most commonly used small datasets in the field of complex and sociological network analysis. The scenario represented is that of a karate club being split into two new organizations as a result of a disagreement over pricing between club president John A. and instructor Mr. Hi (pseudonyms). The new club membership aligned along ideological views, and although classes and club meetings would be exclusive, members would still interact outside of the club due to their pre-existing friendships, which were still intact. The network has 2 reference classes with 34 nodes and 78 links.
2. Dolphin network (Dolphin Social Network) was built by Lusseau *et al.* [18]. It is a relation-network between bottlenose dolphins. Individuals live in large, mixed-sex groups in which no permanent emigration/immigration has been observed over the past 7 years. Though strong associations occur within and between the sexes, there are no clear sub-units existing in the community. Long-lasting associations are a strong feature of the community structure and this stability in the dynamics of association was observed within and between the sexes. Each node represents a dolphin and each link represents close contact between each of the two linked dolphins. The network has 2 reference classes with 62 nodes and 159 links.
3. US Politics network (Books about US politics) [19] is a network of political books sold on the Amazon and compiled by Krebs. In this network the nodes represent 105 recent books on American politics bought through the on-line bookseller Amazon.com, and links join pairs of books that are frequently purchased by the same buyer. Krebs divided the books according to their stated or apparent liberal or conservative alignment. There were however a small number of books that were explicitly bipartisan or centrist, or had no clear affiliation, therefore Newman defined three node classes called “liberal”, “neutral”, and “conservative” [21]. The network has 3 reference classes with 105 nodes and 441 links.
4. Football network (American College Football) [1] is a dataset containing the schedule the games had during the 2000 college football season. The nodes represent the individual football team and the links represent the regular season games between two teams. The teams are divided into 12 “conferences” containing between 8 to 12 teams each. Games are more frequent between members of the same conference than between members of different conferences, while inter-conference play is not uniformly distributed. Teams that are geographically close to one another but belong to different conferences are more likely to play against each other than teams separated by large geographic distances. The network has 12 reference classes with 115 nodes and 615 links.
5. Y2H (yeast two-hybrid) [20] is a network of *Protein-protein Interactions* (PPI), and was obtained by high-throughput yeast two-hybrid screening. It was proposed by Yu *et al*. in 2008. An empirically-controlled mapping framework has been developed to produce a “second-generation” high-quality, high-throughput Y2H data set covering approximately the 20% of all yeast binary interactions. Both Y2H and affinity purification followed by mass spectrometry (AP/MS) data are of equally high quality, but of a fundamentally different and complementary nature, resulting in networks with different topological and biological properties. The union of Uetz-screen, Ito-core, and CCSBYI1 as a “Y2H-union” contains 2930 binary interactions among 2018 proteins. After reducing redundancy nodes and small isolated sub-networks, 1647 nodes and 2518 links are left for further experimentation. The network has 3 sources with 1647 nodes and 2518 links.
6. Artificially-Generated networks [1], [10], are further used to compare the capabilities of ELC and other methods. The artificially-generated networks were randomly generated with an experimental setting similar to the one used by Newman in 2002 [1] and Guimera in 2005 [10]. Each artificially-generated dataset has 128 nodes divided into 4 communities with 32 nodes each. The links are generated using the following definitions with respect to the desired average degree and the proportion of community inside links. Let the average degree of the whole network be 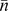 and the proportion of community inside links be 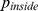, then the proportion of outside links between different communities 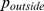
will be 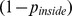, with 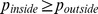 for reasonable communities. The generation procedure places 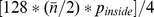 links connecting node pairs chosen at random within each individual community with the constraint that there exists a connected sub-tree. Then it randomly puts the remaining 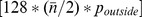 links as outside links for the nodes in different communities. In the experiments, we are not only using different 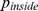 proportions, but also setting different node average degrees 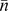 to simulate a range of real-world networks with varying situations. The 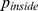 proportion is adjusted from 0.9 to 0.5 by -0.1 steps, and meanwhile the node average degrees is set to 4, 8 and 12, producing 15 conditions with distinct pairs of values. We would expect that the disruptive overlapping between different communities may increase as the node average degree grows and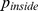 drops. Under each condition, we generated 10 networks and the result values are the average over the 10 instances. All the artificially-generated networks have 4 known classes with 128 nodes, and different artificial topologies with 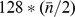 links.

### Link Clustering Algorithm

Evans and Lambiotte first proposed the line graph for detecting an overlapping community structure in networks by links instead of nodes in 2009 [7]. The following year, Ahn *et al.* implemented the same idea by using the Jaccard link similarity and proposed *Link Clustering* (LC) [8] as an alternative method. LC first calculates the link similarity of the neighbor links and then builds a transform matrix, which is then subjected to a hierarchical clustering technique to generate a dendrogram. By calculating the partition density of each level of the dendrogram, the maximum density value can be determined and used to determine the appropriate cutoff level of the dendrogram. The resulting communities are the communities detected.

#### Link similarity

Considering an undirected and unweighted network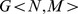, where *N* is the set of nodes in the network, and *M* is the set of the links, let 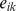 represent the link that connects nodes *i* and *k*. We call two links “neighbor links” if they connect a common node. For the neighbor links 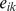 and 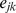 which have a common connected node *k*, the link similarity [8] is the Jaccard distance [23], written as *LS* for short:
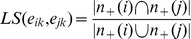
(1)where 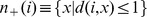 is the *inclusive* neighbor nodes set of node *i*, which contains the node *i* itself and its neighbors, and 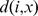 is the length of the shortest path between nodes *i* and *x*.

**Figure 1:**
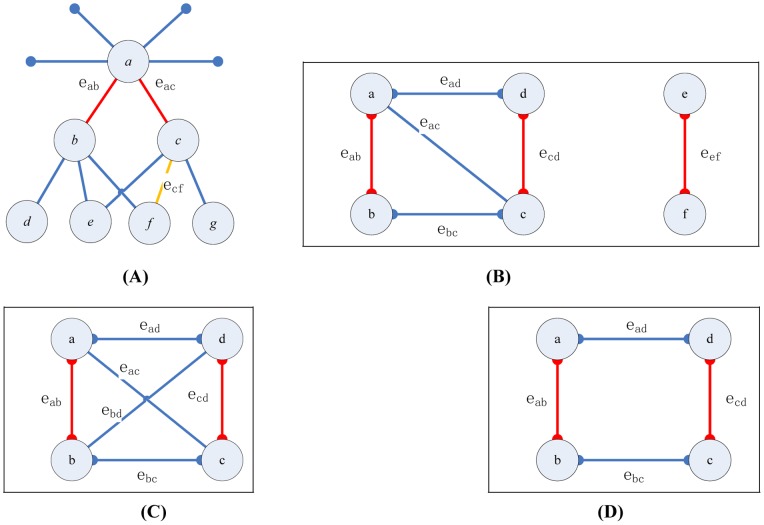
Examples for link similarity calculation. (**A**) A simple example for the link similarity calculation. (**B**) First example to show the limitation of the original link similarity calculation. (**C**) Second example to show the limitation of the original link similarity calculation. (**D**) Third example to show the limitation of the original link similarity calculation.

As shown in the example in Figure 1(A), the intersection between the neighbor nodes of the nodes *b* and *c*, contains *a*, *e* and *f*, and their union contains *a*, *b*, *c*, *d*, *e*, *f* and *g*, so the neighbor link similarity 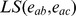 is 3/7≈0.43. According to the definition of link similarity, if two links have no common neighbor nodes, then their link similarity is 0, as depicted in Figure 1(A) for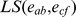.

#### Link clustering procedure

The link clustering procedure is comprised of two main phases as hierarchical clustering on link similarity transform matrix and dividing the dendrogram, which are described below.

According to formula (1), we can get a transform matrix *S* after calculating link similarity between all links in the network. Denoting the cardinality of the set of links *M* in the network as 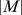, the transform matrix *S* is a square matrix of dimensions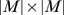. We can define each element
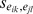
of *S* as follows.
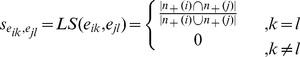
(2)


As can be seen, the elements in the transform matrix are computed by the similarity of neighbor links. To determine clusters on the matrix *S*, we used single-linkage hierarchical clustering that is a general bottom-up clustering technique applicable to any set of elements. The linkage refers to the aggregation that is to be iteratively applied between the clusters showing minimal distance or maximal similarity. With single-linkage, the distance (or similarity) of a pair of clusters is computed as the minimal distance (or maximal similarity) of the pairs of elements across the two clusters. Consequently, a single pair of elements determines the aggregation of two clusters. The application is described in the following steps:

1. Initialize every link as a singleton cluster and compute their similarity.
2. Select the pair of clusters with the maximal similarity (namely the maximal link similarity between pairs of links across the clusters) and aggregate them into a new cluster and compute its similarity with the other clusters.
3. Repeat step 2 until all links merge into one cluster.
4. Output a dendrogram.

To determine the best cutting level for the dendrogram, *LC* uses the partition density calculation. This calculation is defined as follows.

Given a cutting level for the dendrogram, let *C* be the corresponding collection of subsets of *M* (set of links in the network) that represents the covering of *M* by *k* communities, namely 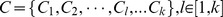. The number of links in the subset 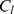 is 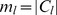. The number of nodes connected by the links of 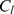 is 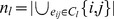


The definition of a single community’s density is
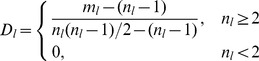
(3)


The definition of a partition density for the cutting level is
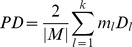
(4)


The partition density PD is the function of the cutting level for the dendrogram, and the best cutting level is the solution that maximizes the PD. Once all the partition densities have been computed, we can choose the level with the maximal *PD* value to divide the network and get the division of communities in terms of a partition of links. Though the resulting partition contains non-overlapping link clusters, some links belonging to different clusters may have common connected nodes in the original network. The link clusters naturally determine the final division results for the nodes in the network with corresponding node communities that can be overlapped.

#### Limitation of link clustering algorithm

Although LC has many advantages in determining overlapping community detection, the original link similarity (based on the Jaccard distance calculation) discharges part of the information between links during the matrix transformation. In fact, the similarity only considers the neighbor links, namely those with common nodes, and neglects the similarity between non-neighbor links. The lost information may influence the results of the community analysis. As shown in Figure 1(B), the original link similarity 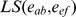
of links 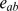 and 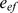 is 0, but the similarity 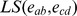 of 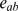 and 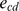 is also 0, since 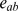
and 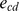 have no common neighbor nodes. Clearly, the links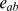 and 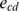 should belong to the same community and the similarity 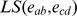 between them should be higher than that of 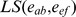. This demonstrates a potentially important misrepresentation of some aspects of the structure that may be relevant for clustering and analysis; hence, it can potentially influence the results of the division of communities.

With regard to the partition density, we tend to observe a division of the communities into small communities as a result of the hierarchical clustering. Consider Figure 1(B), where three communities with two overlapped triangles has a partition density of 1, while for two communities, the partition density is 0.56. However, it seems that 2 communities should be more reasonable and thus carry the higher value PD, which is not the case in this example. Formulas (3) and (4) clearly show that for increasing values of *n_l_*, the value of 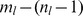 will exhibit a slower increase in value than that of 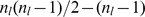, thus resulting in the network being divided into smaller communities.

### Extended Link Clustering Algorithm

#### Extended link similarity

Given the limitation of the original link similarity method, we propose a new *Extended Link Similarity* (ELS) defined below.
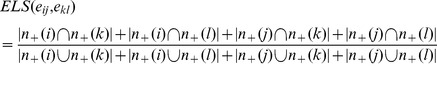
(5)


For the links 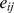 and 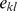, 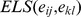 calculates the ratio of the sum of the cardinalities of the intersection sets of nodes and the sum of the union sets of nodes connected by the links. Not only does *ELS* consider neighbor link similarity, but it also introduces the non-neighbor links similarity in the calculation. With more information about the relationship between links now available, we can get a denser transform matrix for better clustering and community analysis. Using the same network shown in Figure 1(B), 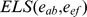 still equals 0, since 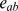 and 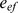 has no intersection nodes and has no relationships at all. However, 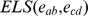 will now achieve a more realistic value of 0.75, which is greater than 0 and represents the existence of indirect links that are now considered in *ELS*.

The link similarity and the extended link similarity methods convey different degrees of information. For example, in each of the quadrangle structures shown in Figure 1(B), Figure 1(C) and Figure 1(D), the values of extended link similarity of 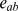 and 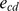 are different (0.75, 1 and 0.5 respectively), varying with the number of indirect links. The values of the link similarity method are still 0 however, and are independent of the structure having more indirect links. In short, ELS is more representative of links within a real community network when non-neighbor/indirect links are of substantive value.

The link similarity and the extended link similarity convey different information. The link similarity conveys less information of links structure. In each of the quadrangle structures shown in Figure 1(B), Figure 1(C) and Figure 1(D), the values of extended link similarity of 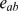 and 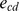 are different, respectively 0.75, 1 and 0.5 varying with the number of indirect links. But the values of link similarity are still 0 and do not change with the structure having more indirect links.

#### EQ evaluation division

To avoid the shortcomings of the partition density division, and to arrive at better cut level decisions, we propose using EQ evaluation instead. A quality function “modularity” Q was proposed by Newman *et al*. for the evaluation of the communities subsequent to proposing the use of maximal modularity [2]. An extension of modularity EQ was introduced to evaluate the “goodness” of overlapped community decomposition by Shen *et al*. in 2009 [16].

The definition 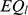 of a single community is
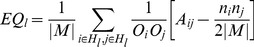
(6)where *H_l_* represents a community node set after the division of the network into k communities. *M* is the set of links in the network and 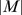 is the total number of links in the network. 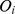 represents the number of communities that node *i* belongs to. If there is a link between node *i* and node *j*, the value 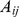 is 1; otherwise the value 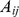 is 0. 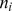 is the degree of node *i*.

The *EQ* of the whole communities is calculated as shown below.
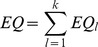
(7)


The higher the *EQ* value, the more reasonable the overlapping communities are.

For the same network shown in Figure 1(B), the *EQ* value is 0.1285 with three communities, and it will have a higher value 0.2361 with two communities, which is more sensible.

#### Extended link clustering procedure

Based on the extended link similarity definition formula (5) and the EQ community evaluation, we propose the extended link clustering (ELC) method. The procedure of the ELC method is based on the original LC. The following steps describe this new proposed method.

1. Compute the transform matrix *S* after calculating link similarity between all links in the network according to the ELS formula (5).
2. Use the single-linkage hierarchical clustering method to get the dendrogram.
3. Calculate EQ values according to the formula (6) for each level of the dendrogram and cut it at the level having the maximal EQ value.

**Figure 2:**
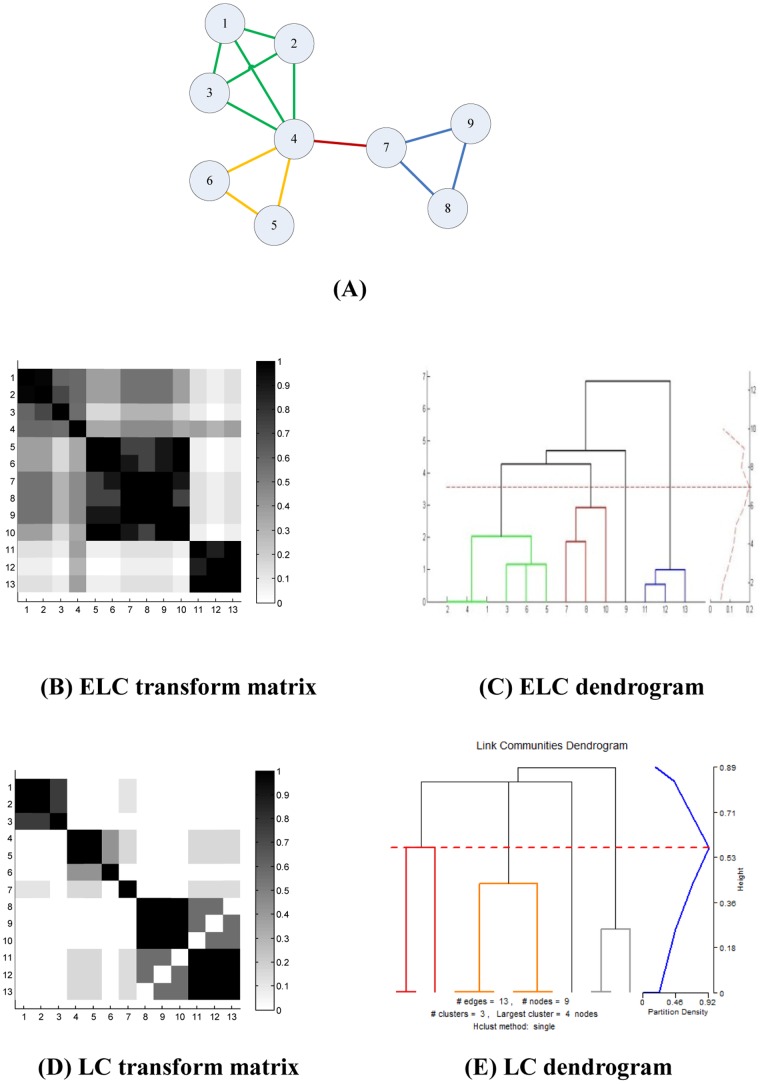
A simple network for ELC and LC calculation. (**A**) A simple network example mentioned in Ahn’s paper (2010). (**B**) The transform matrix and (**C**) The dendrogram obtained by ELC on (A)’s example networks. (**D**) The transform matrix and (**E**) the dendrogram obtained by LC on (A)’s example networks.

For the network shown in Figure 2(A), which was mentioned in Ahn’s paper [8], the dendrogram produced by the ELC method is shown in Figure 2(C); with the corresponding graph produced by the original LC method shown in Figure 2(E). Now, comparing Figure 2(B) and Figure 2(D), it can be seen that ELC can achieve a denser transform matrix that is richer in information and potentially better for cluster analysis, even though both methods produce the same communities results in this very simple network example.

### Evaluation Procedure

For experimental evaluation, we ran ELC against all datasets previously mentioned, and we compare our results to LC and CPM, which is a classic node-based method for the analysis of community structure. Here, we use the R project package “linkcomm” [14] (version 1.0.6) that implements the LC method and the CFinder’s package [5] (version2.0.5), which provides us with the faction filtering algorithm CPM.

Before evaluating the overlapping communities’ results, we should emphasize that LC and CPM methods may not map every node in a network dataset to that of an identified community. This can result from the CPM algorithm filtering out too many nodes during its execution. To compensate for this, we also calculate the cover rate (covered nodes/all nodes) and the number of uncovered nodes for real-world datasets. In our runs the complete sub-graphs (size *k*) of *k*-clique in the CPM method is set to 3 or 4, which provisions the final results to be much closer to the real community numbers.

Since EQ is the measure for dendrogram cutoff decisions in ELC, and while the partition density is used for cutoff decisions in LC, we adopt a third evaluation measure called *In-group Proportion* (IGP) to assess the communities produced by the different methods. IGP is a measure of cluster quality proposed by Kapp *et al*. in 2007 [15], and is based on the concept of prediction accuracy. It is defined to be the proportion of nodes in a group whose nearest neighbor is also in the same group. Suppose the whole network *G* is divided into *k* communities 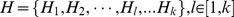. The IGP value of community *H_l_* can be calculated by formula (8):
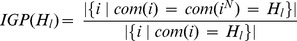
(8)where 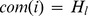 indicates that node *i* belongs to community *H_l_*. For node *i*, 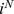
is the *i*’s nearest neighbor node, and 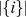
denotes the number of nodes meeting the condition. We can describe 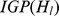 as the proportion of nodes in community 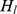 whose neighbor nodes are also in community 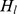 [15]. Finally, we can get the 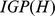 of all the communities by formula (9) as follows.



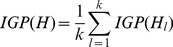
(9)In order to analyze the Y2H networks results further, we also computed the Gene Ontology (GO) enrichment, which has been widely used in bioinformatics in recent years [24]. In general, the genes in most communities are suitably annotated to reliable functions on three GO categories, i.e., molecular functions, biological process and cellular component. The corresponding GO category p-values are probabilities of the null hypothesis enrichment, and so they range between 0 and 1. The closer the p-value is to zero, the more significant the particular GO term associated with the group of genes. In the following experiments, we use BINGO version 2.44 [25], which is a plugin for Cytoscape [26] to evaluate the GO enrichment performance.

In summary, in the experimental data analysis, we use EQ value, the partition density, IGP measure, communities number (CN), cover rate (CR), and uncovered nodes (UN) to evaluate the overlapping communities’ quality across all our five real-world networks and several artificially-generated networks. We also applied GO enrichment to enhance the analysis of the Y2H (yeast two-hybrid) dataset further.

## Results

**Figure 3:**
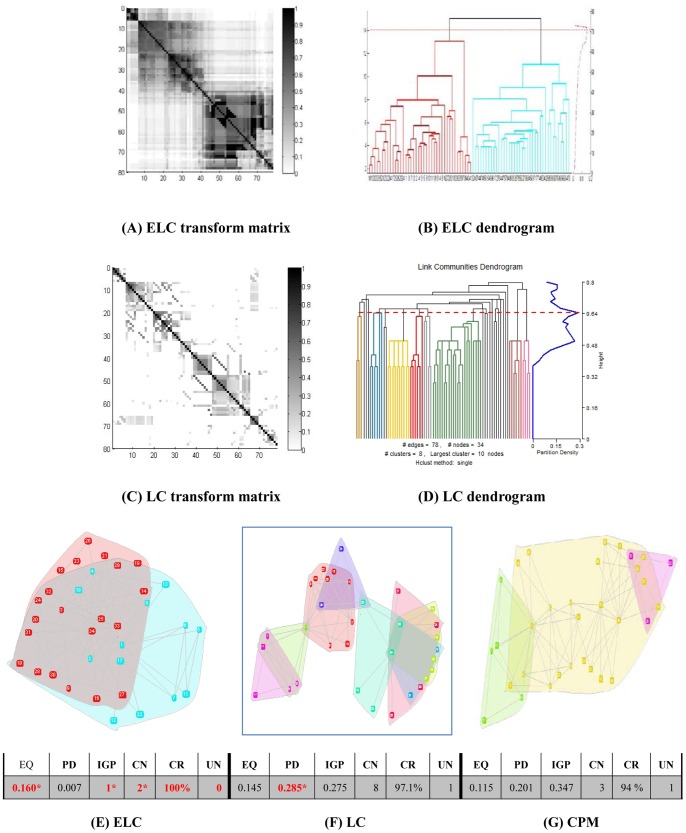
Karate network (34 nodes/2 classes). The transform matrix (**A**) and the dendrogram (**B**) obtained by ELC, the transform matrix (**C**) and the dendrogram (**D**) obtained by LC. (**E–G**) Communities and corresponding values of Extended Quality of modularity (EQ), Partition Density (PD), In-Group-Proportion (IGP), Communities Number (CN), Cover Rate (CR) and number of Uncovered Nodes (UN) obtained by ELC, LC and CPM. *the red and bold data marked with an asterisk (*) is the best value of each evaluation on the dataset for the three methods.

**Figure 4:**
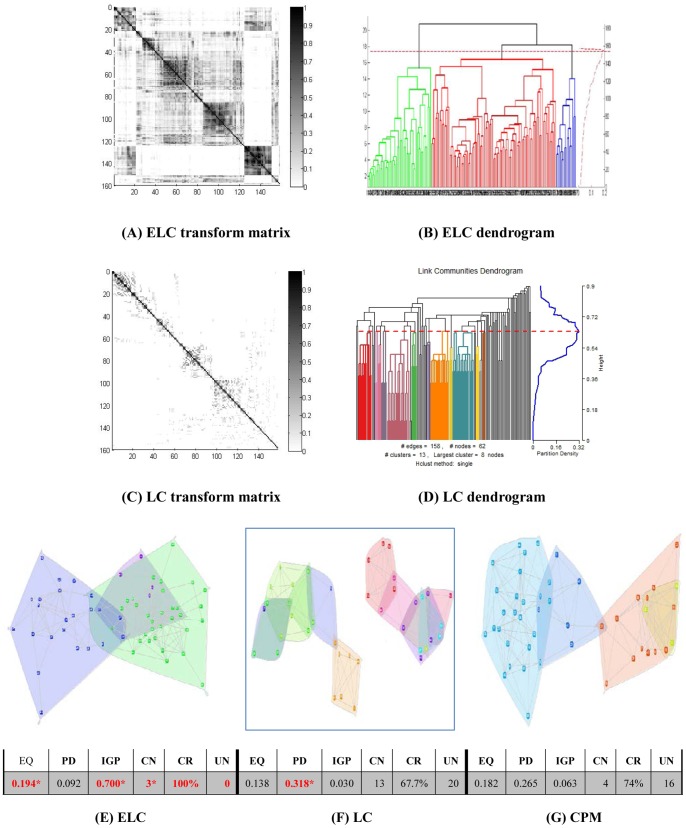
Dolphin network (62 nodes/2 classes). The transform matrix **(A)** and the dendrogram (**B)** obtained by ELC, the transform matrix **(C)** and the dendrogram **(D)** obtained by LC. **(E-G)** Communities and corresponding values of Extended Quality of modularity (EQ), Partition Density (PD), In-Group-Proportion (IGP), Communities Number (CN), Cover Rate (CR) and number of Uncovered Nodes (UN) obtained by ELC, LC and CPM. *the red and bold data marked with an asterisk (*) is the best value of each evaluation on the dataset for the three methods.

**Figure 5:**
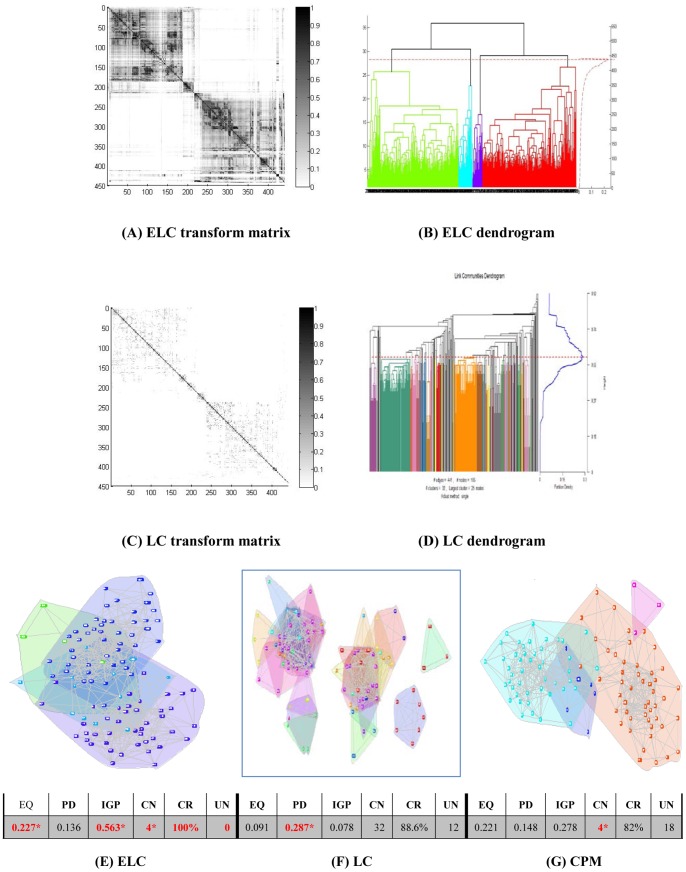
US politics network (105 nodes/3 classes). The transform matrix (**A**) and the dendrogram (**B**) obtained by ELC, the transform matrix (**C**) and the dendrogram (**D**) obtained by LC. (**E–G**) Communities and corresponding values of Extended Quality of modularity (EQ), Partition Density (PD), In-Group-Proportion (IGP), Communities Number (CN), Cover Rate (CR) and number of Uncovered Nodes (UN) obtained by ELC, LC and CPM. *the red and bold data marked with an asterisk (*) is the best value of each evaluation on the dataset for the three methods.

**Figure 6:**
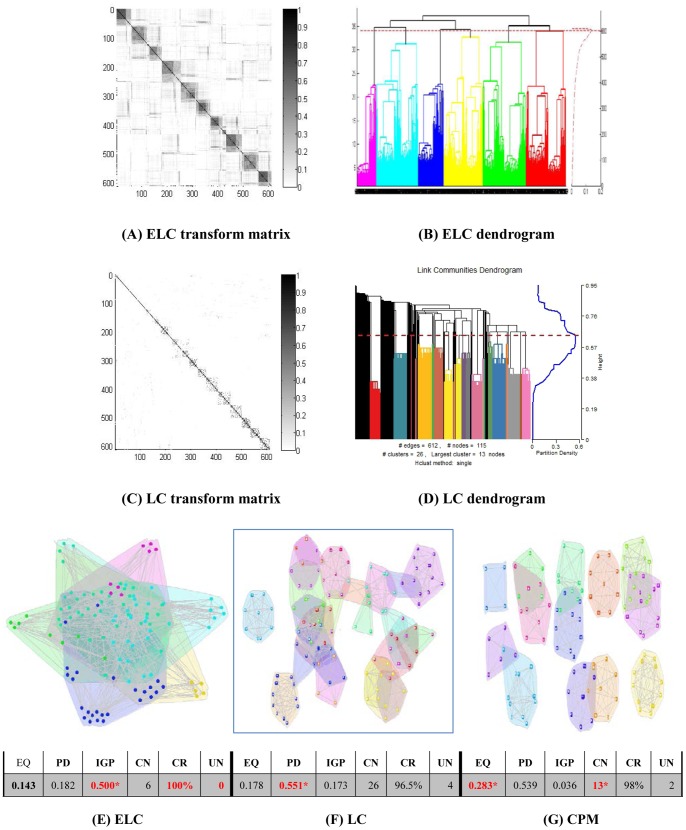
Football network (115 nodes/12 classes). The transform matrix (**A**) and the dendrogram (**B**) obtained by ELC, the transform matrix (**C**) and the dendrogram (**D**) obtained by LC. (**E–G**) Communities and corresponding values of Extended Quality of modularity (EQ), Partition Density (PD), In-Group-Proportion (IGP), Communities Number (CN), Cover Rate (CR) and number of Uncovered Nodes (UN) obtained by ELC, LC and CPM. *the red and bold data marked with an asterisk (*) is the best value of each evaluation on the dataset for the three methods.

**Figure 7:**
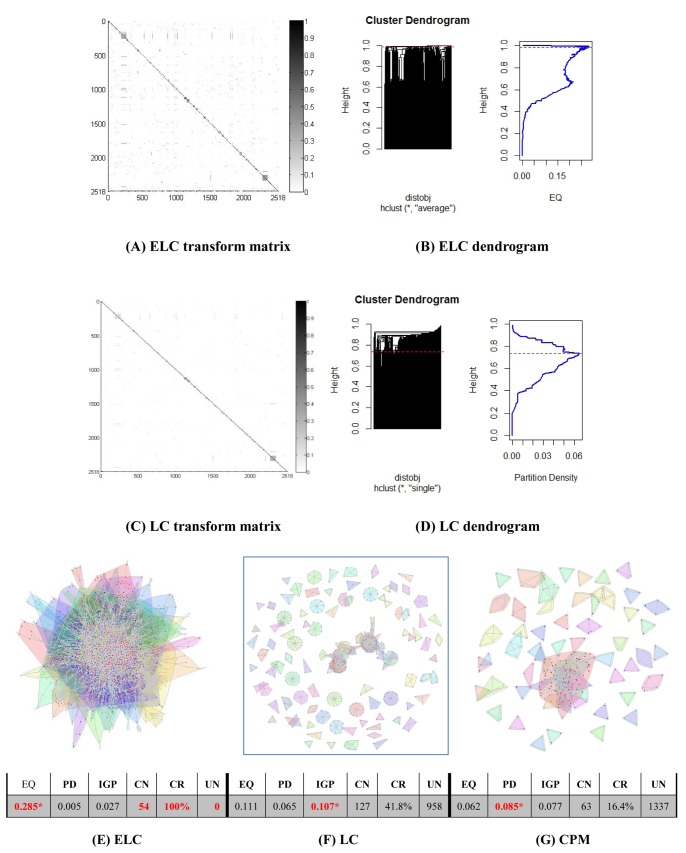
Y2H network (1647 nodes/3 sources). The transform matrix (**A**) and the dendrogram (**B**) obtained by ELC, the transform matrix (**C**) and the dendrogram (**D**) obtained by LC. (**E–G**) Communities and corresponding values of Extended Quality of modularity (EQ), Partition Density (PD), In-Group-Proportion (IGP), Communities Number (CN), Cover Rate (CR) and number of Uncovered Nodes (UN) obtained by ELC, LC and CPM. *the red and bold data marked with an asterisk (*) is the best value of each evaluation on the dataset for the three methods.

**Figure 8:**
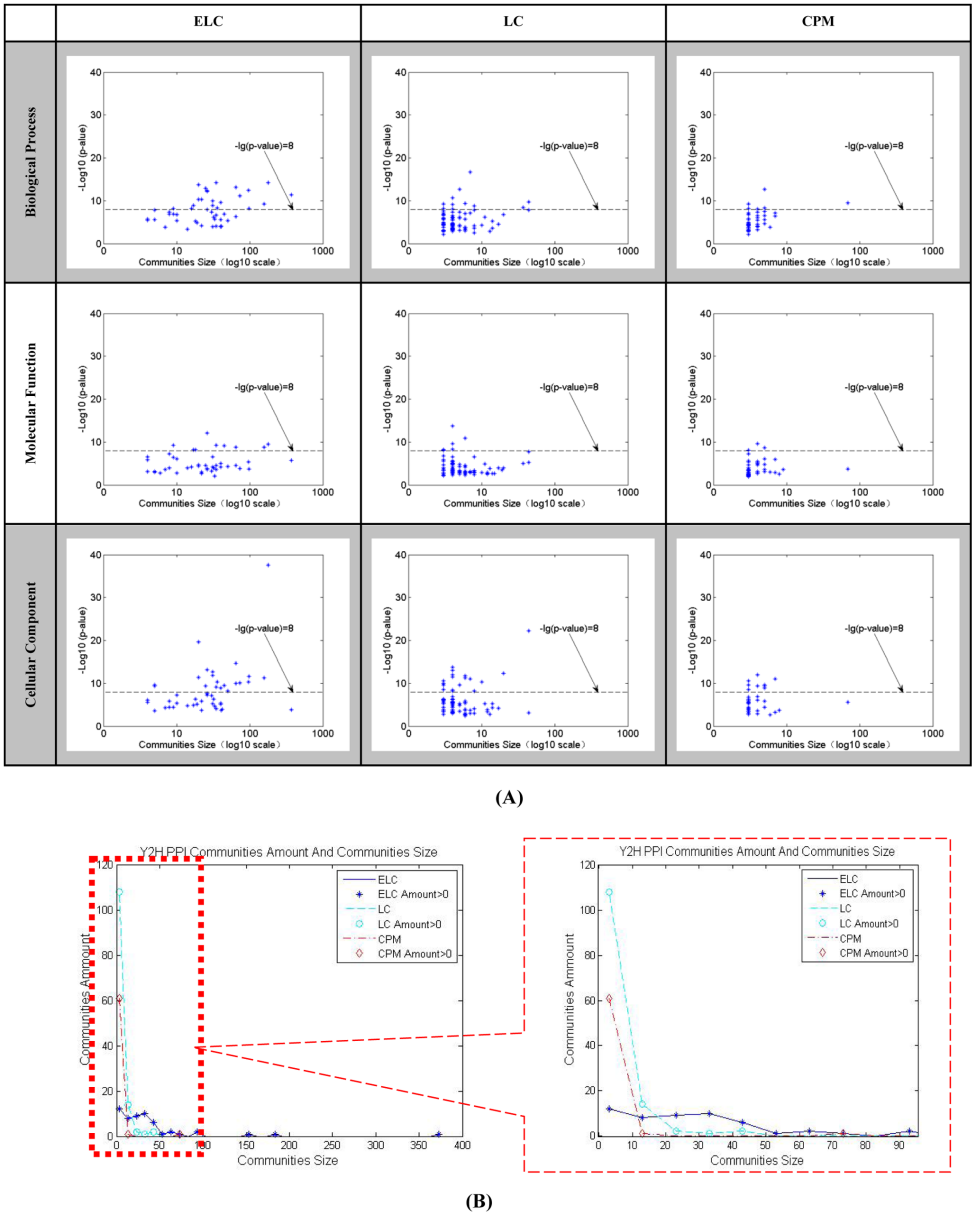
Y2H network for GO enrichment analysis. (**A**) Y2H network’s community numbers and GO enrichment values obtained by ELC, LC and CPM. Axis x is log10 community numbers and axis y is –log10 p-values of all modules GO enrichment for biological process, molecular functions and cellular component. The average communities size found by ELC are much higher than LC and CPM by GO categories at smaller p-value level, especially when p-values are lower than E-8. (**B**) Y2H network’s statistics on nodes number of communities by ELC, LC and CPM.

**Figure 9:**
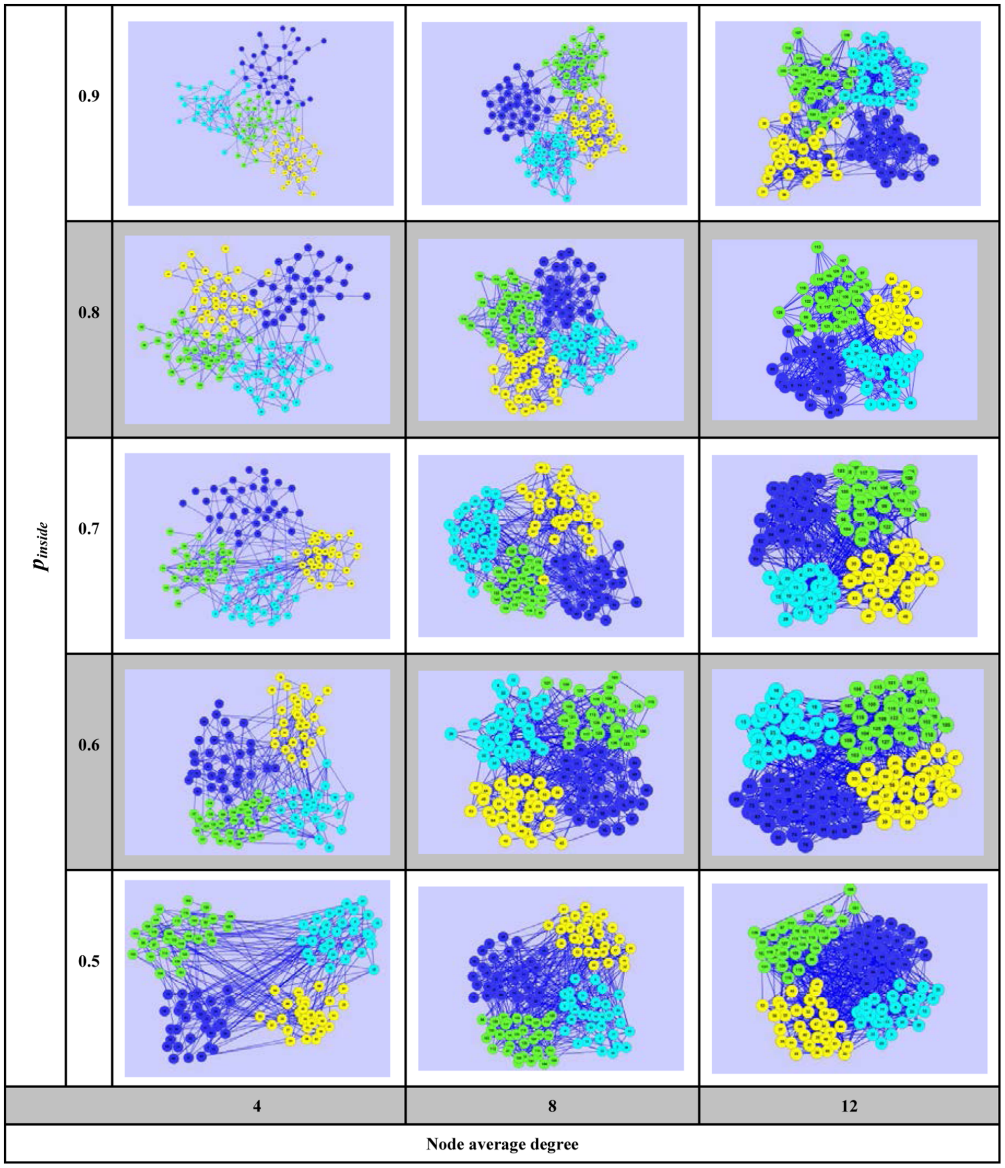
A selected artificial network set with different node average degrees and ***p_inside_*** **values.**

Results on the real world datasets are presented in Figures 3–7. Each figure is devoted to a single dataset and it is comprised of the transform matrices and dendrograms of ELC and LC, the communities found by them and by CPM, and the corresponding values of EQ, PD, IGP, CN, CR and UN. The results of the GO enrichment analysis on Y2H are presented in Figure 8 and Table 1. The results of the analysis on the artificially-generated network are shown in Figure 9 and Tables 2–4. Finally, all the measures of 5 real world datasets are also collected for direct comparison in Tables 5 and 6. In the following sections, we will present the details of the results of each evaluation.

**Table 1:** Proteins number (PN) in the top 10 communities of three methods sorted by GO enrichment values ranked p-values of all modules for biological process, molecular functions and cellular component.

ELC Method						LC Method						CPM Method					
BiologicalProcess		MolecularFunctions		CellularComponent		BiologicalProcess		MolecularFunctions		CellularComponent		BiologicalProcess		MolecularFunctions		CellularComponent	
PN	p-value	PN	p-value	PN	p-value	PN	p-value	PN	p-value	PN	p-value	PN	p-value	PN	p-value	PN	p-value
179	5.30E-15	26	7.43E-13	179	**2.96E-38** *	7	**1.85E-17** *	4	**1.71E-14** *	44	5.11E-23	5	2.02E-13	4	2.16E-10	4	1.14E-12
35	6.14E-15	179	3.07E-10	20	2.50E-20	5	2.02E-13	6	1.19E-11	4	1.63E-14	69	3.07E-10	5	2.34E-09	7	9.23E-12
20	1.79E-15	9	5.90E-10	65	2.22E-15	4	2.21E-11	4	2.16E-10	4	8.13E-14	3	5.02E-10	3	7.48E-09	3	2.52E-11
65	6.64E-14	35	6.15E-10	26	6.27E-14	44	1.95E-10	4	4.27E-09	20	5.23E-13	5	5.01E-09	3	5.76E-08	3	2.52E-10
25	1.31E-13	45	7.68E-10	31	2.10E-13	6	4.28E-10	3	5.76E-09	4	1.14E-12	3	5.52E-09	5	9.60E-07	5	2.52E-10
96	3.76E-13	65	1.67E-09	31	1.25E-12	3	5.02E-10	3	7.48E-09	6	1.87E-12	4	9.13E-09	7	1.12E-06	4	4.03E-10
26	4.64E-13	156	1.80E-09	98	2.61E-12	3	5.02E-10	44	1.64E-08	4	3.42E-12	5	3.27E-08	3	2.40E-06	5	4.46E-10
26	6.93E-13	18	5.78E-09	20	3.87E-12	4	7.50E-10	3	1.60E-07	6	5.17E-12	4	4.13E-08	4	3.20E-06	5	1.21E-09
373	4.13E-12	17	6.74E-09	156	5.82E-12	8	1.52E-09	3	1.60E-07	7	9.23E-12	3	7.34E-08	3	4.48E-06	3	3.02E-09
73	6.31E-12	8	5.12E-08	36	4.07E-11	37	4.03E-09	8	2.48E-07	3	2.52E-11	7	7.36E-08	4	5.93E-06	4	1.13E-07

* the bold data marked with an asterisk (*) is the best value of each GO enrichment category.

**Table 2:** ELC performance on different artificial datasets conditions.

Average degree	4				8				12			
*p_inside_*	EQ	PD	IGP	CN	EQ	PD	IGP	CN	EQ	PD	IGP	CN
**0.9**	**0.291** *	0.046	**0.347** *	4.3	0.247	0.138	**0.393** *	4.4	0.205	0.138	0.329	5.2
**0.8**	**0.227** *	0.034	**0.260** *	5.8	0.151	0.117	**0.395** *	4.6	0.114	0.135	0.421	5.4
**0.7**	**0.183** *	0.026	**0.227** *	8.3	**0.093** *	0.082	**0.455** *	5.5	0.059	0.130	**0.318** *	7.8
**0.6**	**0.173** *	0.024	**0.175** *	7.7	0.070	0.071	**0.375** *	7.7	**0.033** *	**0.156** *	0.349	10.1
**0.5**	**0.159** *	0.024	**0.164** *	9.0	0.057	0.065	**0.270** *	10.4	**0.026** *	**0.204** *	0.402	12.9

* the bold data marked with an asterisk (*) is the best value with the same location in Tables 2–4.

** EQ: Extended Quality of modularity; IGP: In-Group-Proportion; PD: Partition Density; CN: Communities Number.

To avoid accidental influence of single artificial network, all types of evaluation values are average values of 10 networks in each condition.

**Table 3:** LC performance on different artificial datasets conditions.

Average degree	4				8				12			
*p_inside_*	EQ	PD	IGP	CN	EQ	PD	IGP	CN	EQ	PD	IGP	CN
**0.9**	0.118	**0.319** *	0.056	17.1	**0.295** *	**0.839** *	0.224	7.4	**0.289** *	**0.616** *	0.146	7.9
**0.8**	0.111	**0.436** *	0.096	16.8	0.148	**0.452** *	0.071	21.6	**0.177** *	**0.586** *	0.098	17.3
**0.7**	0.086	**0.327** *	0.046	14.0	0.088	**0.152** *	0.109	27.1	**0.090** *	**0.557** *	0.118	28.8
**0.6**	0.086	**0.295** *	0.069	16.1	0.078	**0.260** *	0.095	31.4	0.016	0.108	**0.725** *	14.4
**0.5**	0.087	**0.284** *	0.061	15.5	0.064	**0.208** *	0.095	33.7	0.004	0.114	**0.901** *	1.0

* the bold data marked with an asterisk (*) is the best value with the same location in Tables 2–4.

** EQ: Extended Quality of modularity; IGP: In-Group-Proportion; PD: Partition Density; CN: Communities Number.

To avoid accidental influence of single artificial network, all types of evaluation values are average values of 10 networks in each condition.

**Table 4:** CPM performance on different artificial datasets conditions.

Average degree	4				8				12			
*p_inside_*	EQ	PD	IGP	CN	EQ	PD	IGP	CN	EQ	PD	IGP	CN
**0.9**	0.101	0.221	0.037	17.3	0.274	0.173	0.162	7.3	0.201	0.175	**0.640** *	3.0
**0.8**	0.088	0.189	0.083	15.1	**0.181** *	0.178	0.099	15.8	0.028	0.090	**0.661** *	2.2
**0.7**	0.067	0.151	0.063	12.3	0.091	0.172	0.103	21.8	0.007	0.094	0.307	4.6
**0.6**	0.066	0.158	0.077	12.6	**0.087** *	0.210	0.061	39.8	0.011	0.107	0.245	7.8
**0.5**	0.051	0.120	0.030	10.0	**0.075** *	0.203	0.118	30.0	0.016	0.131	0.221	13.8

* the bold data marked with an asterisk (*) is the best value with the same location in Tables 2–4.

** EQ: Extended Quality of modularity; IGP: In-Group-Proportion; PD: Partition Density; CN: Communities Number.

To avoid accidental influence of single artificial network, all types of evaluation values are average values of 10 networks in each condition.

**Table 5:** Comparison with three methods on five real-world networks by four different evaluations.

Dataset(nodes/classes)	ELC				LC				CPM			
	EQ	PD	IGP	CN	EQ	PD	IGP	CN	EQ	PD	IGP	CN
**Karate(34/2)**	**0.160** *	0.007	**1** *	**2** *	0.145	**0.285** *	0.275	8	0.115	0.201	0.347	3
**Dolphin(62/2)**	**0.194** *	0.092	**0.700** *	**3** *	0.138	**0.318** *	0.030	13	0.182	0.265	0.063	4
**US politics(105/3)**	**0.227** *	0.136	**0.563** *	**4** *	0.091	**0.287** *	0.078	32	0.221	0.148	0.278	**4** *
**Football(115/8-12)**	0.143	0.182	**0.500** *	6	0.178	**0.551** *	0.173	26	**0.283** *	0.539	0.036	13*
**Y2H (1647/3)**	**0.285** *	0.005	0.027	**54**	0.111	0.065	**0.107** *	127	0.062	**0.085** *	0.077	63

* the bold data marked with an asterisk (*) is the best value of each evaluation on the dataset for three methods.

** EQ: Extended Quality of modularity; IGP: In-Group-Proportion; PD: Partition Density; CN: Communities Number.

**Table 6:** Comparison with three methods on five real-world networks by cover rate and uncovered nodes.

Dataset(nodes/classes)	ELC		LC		CPM	
	CR	UN	CR	UN	CR	UN
**Karate(34/2)**	**100%**	**0** *	97.1%	1	94%	1
**Dolphin(62/2)**	**100%**	**0** *	67.7%	20	74%	16
**US politics(105/3)**	**100%**	**0** *	88.6%	12	82%	18
**Football(115/12)**	**100%**	**0** *	96.5%	4	98%	2
**Y2H (1647/3)**	**100%**	**0** *	41.8%	958	16.4%	1337

* the bold data marked with an asterisk (*) is the best value of each evaluation on the dataset for three methods.

** CR: Cover Rate; UN: number of Uncovered Nodes.

### Karate Dataset Results

From Figure 3(A) and Figure 3(C), we can visually see how the transform matrix computed by ELC is denser than the one produced by the original LC method. There are two obvious communities in Figure 3(A), but the blocks in LC’s transform matrix are less apparent. From Figure 3(E) and Figure 3(F), the ELC method identified 2 communities with an EQ value of 0.160, while the LC method identified 8 communities with an EQ value of 0.145. The LC method produced smaller communities and did not achieve the expected real world representation. Additionally, 1 node was left uncovered in the final results. From Figure 3(G), we can see that the CPM method identified 3 communities and its EQ value was 0.115, which is lower than the ELC method’s EQ value. Since the CPM method tends to find the biggest block in the network, it left two nodes uncovered in the final results.

In Figure 3(E), nodes set (3,9,10,14,20,28,29,31,32) construct the overlapping part in the ELC communities. We can see that these 9 nodes are located in the adjacent area of the two communities. From Figure 3(F) and Figure 3(G), there are also some overlap nodes in different communities, but the overlapping areas are all very small. At the same time, the IGP of ELC achieved a perfect value of 1, which indicates that all the nodes have their own nearest neighbor in the same community.

### Dolphin Dataset Results

From Figure 4(A) and Figure 4(C), we can see that the transform matrix generated from LC is unclear and not that informative, while the ELC transform matrix clearly represents a network divided into three big clusters. Moreover, the EQ value is 0.194, and the number of communities corresponds with the number of communities mentioned in the original dataset paper [18]. LC identified 13 communities with EQ value of 0.138, with the biggest community having only 8 members, which is far from the original research paper results [18]. The CPM method identified 4 communities with EQ value of 0.182, and although it is very close to the ELC’s division results, CPM discharges 16 nodes resulting in only a 74% cover rate.

In Figure 4(E), the final three communities found by ELC have 10, 16 and 36 nodes respectively. The overlapping part contains individual dolphins (Zipfel, TR99, TR77, Thumper, SN89, SN100, PL, Oscar, DN63) that communicate with different dolphins in other regions. ELC also attained the best value of IGP and it shows that the communities found by ELC have a greater number of nearest neighbors than the other methods.

### US Politics Dataset Results

From Figure 5(A) and Figure 5(C), we can see that the transform matrix computed by the LC method is relatively sparse, and its blocks are relatively obscure. On the other hand, ELC generates a transform matrix that clearly shows two big communities. The ELC method identified 4 communities with an EQ value of 0.228. On the contrary, the LC method identified 32 communities with an EQ value of 0.091. For the CPM method, 4 communities were identified with an EQ value of 0.221. Again, we see that the CPM method performs well in identifying the number of communities, but it does so at the expense of the cover rate, which is only 82% as a result of discharging 18 nodes.

In Figure 5(E–G), the final four communities of ELC have 12, 15, 49 and 55 nodes. Each of the two smaller communities is local (near) to a big community, which is similar to the CPM results. However, the LC method obtains 32 communities with only one large 25-node community and more than 30 communities with less than 10 nodes each. The fact that the best IGP value was reached by ELC demonstrates again that it can place the greatest number of nearest neighbors in the same community.

### Football Dataset Results

From Figure 6(A) and Figure 6(C), we can see that is difficult to distinguish the blocks directly from the transform matrix obtained from the LC method. For the ELC method, we can distinguish almost 10 blocks in the transform matrix. With the ELC method we obtained 6 communities with an EQ value of 0.143. The LC method identified 26 communities with an EQ value of 0.178. The CPM method identified 13 communities and its EQ value was 0.283, higher than the one reached by the ELC and LC methods.

In Figure 6(E–G), the ELC method achieved the lowest EQ value, with the final communities overlapping and containing a large number of nodes. The number of communities identified is 6 and that is well under the benchmark expected number of 12. Arguably, this could be due to the higher node degree and the many relationships between the inside and outside of the communities in this network (discussed in the next section). For these types of networks, ELC tends to divide the datasets into big communities with much more overlapping. The highest IGP value of ELC is indicative of this tendency, since the IGP value is higher when most of the nearest neighbor nodes are in the same community.

### Y2H (Yeast Two-hybrid) Dataset Results

From Figure 7(A) and Figure 7(C), we can again see that the ELC method generates a denser transform matrix than that of the LC method. In Figure 7(E–G), ELC produced 54 communities and was inclusive of all initial 1647 nodes. In contrast, LC method identified 127 communities with 958 nodes that were not covered, which translates into a very low cover rate of 41.8%. The CPM method identified 63 communities, but it also had a high number of uncovered nodes (1337), which represents more than 2/3 of the entire network. Consequently, it has only a 16.4% cover rate and substantially lower than the LC method. Under such unlikely and low cover rates, direct comparison of EQ, PD and IGP on the three methods makes little sense and is deemed to have little informative value.

In order to determine whether the association between the groups of genes has statistical significance, we evaluate all the functional modules identified in the Y2H PPI network in terms of GO enrichment. In our experiments, we considered p-values less than 0.05 for the three GO categories, i.e., biological process (BP), molecular functions (MF) and cellular component (CC). The top 10 GO enrichment results of the three methods for all three categories are shown in Table 1 in descending order of p-values. Our ELC method gets the smallest p-value (p = 2.96e-38) in CC with a group of 179 nodes, while LC method gets the smallest p-values (p = 1.85e-17) in BP and in MF (p = 1.71e-14) with groups of only 4 and 7 nodes. Across all the p-values of the three methods, ELC has 7, 6 and 5 communities in the top 10 results for BP, MF and CC respectively. The number of nodes the ELC method gets in Table 1 is high, and it has only two communities with less than 10 nodes. On the other side, the LC method only has four communities with more than 10 nodes and the CPM method has only one.

We collected all the p-values of the three methods for statistical analysis and are represented in Figure 8. From Figure 8(A), we can see that the average communities size found by ELC are much higher than LC and CPM by GO categories at smaller p-value level, especially when p-values are lower than E-8. From Figure 8(B), the analysis of the protein numbers in the communities, ELC method tends to get more nodes per community than the other two methods, whereas LC and CPM methods have communities with less nodes and higher p-values.

### Artificially-Generated Networks Results

The selected artificial networks under different conditions are displayed in Figure 9. From Figure 9, we can see that a higher average degree corresponds to more connections between different communities under the same proportion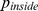. Moreover, when 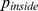 is 0.5, all communities are mixed together and each single community’s outline is not distinct at all. While the ratio reaches 0.9, the networks form four individual communities and tend to have less overlapping.

From Table 2, we can see that when the average degree is 4, regardless of the value of 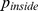, the ELC method always achieves the best average EQ values than the other two methods. When the average degree is 8, ELC method’s results can achieve the best average EQ value at a ratio of 0.7, but still can get the best reasonable average CN values at other ratios. Once the average degree reaches 12, ELC can achieve the best average EQ values and partition density values at the ratio of 0.5 and 0.6. For IGP values, ELC always has the better results, except when the average degree reaches 12.

As Table 3 shows, the LC method always achieves the best average partition density PD value except when the average degree is 12 for a 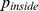 of 0.5 and 0.6. However, one item of interest is that it gets the best average EQ values when the degree is 12 and when the 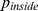 ranges from 0.7 to 0.9. Finally in Table 4, the CPM method only achieves mentionable results for best average EQ values when the average degree is 8.

Across the results from Tables 2–4, we can see that ELC appears to gain more advantage with high 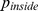 and low average degrees, namely near the top left corner of the corresponding Figure 9. The might be a result of the denser transform matrices it achieved. LC may get better EQ values with high 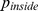 and high average degree, which correlates with the top right corner, while CPM can get reasonable average performance with low values of 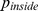, represented by the lower half of Figure 9. This seems to be in line with the fact that LC only considers neighbor links and CPM aims to find the biggest block in the network. As shown, ELC always achieved the best IGP values and consistently kept the nearest neighbor nodes in the same community. It was of no surprise that the LC method exceled in PD values, since it chooses the maximal partition density value to divide the network with many small communities.

## Discussion

From Table 5, which directly compares the five different real-world datasets, we can easily see that ELC always obtained the best IGP values, with the only exception being Y2H as previously discussed. Consequently, ELC tends to put the nearest neighbors in the same community more often than the other methods. Unsurprisingly, LC achieved the best scores in terms of PD values, mainly due to its reliance on the maximal PD value to cut the dendrogram. For a similar reason ELC gets the best EQ values on four of them, with the exception of the Football dataset where it has an EQ lower than the others. In terms of community identification, the ELC method obtained closer results to the benchmark numbers of Karate, Dolphin and Football datasets. Although the US politics dataset does not have a standard benchmark, the 4 communities of ELC are also reasonably comparable with other published’ results [19] [22]. For Y2H network, it’s worth recalling that “Y2H union” comprises data from three different sources: Uetz-screen, Ito-core, and CCSBYI1. This circumstance does not mean that the Y2H network has only three communities; instead the network has lots of “hubs” that appear to be locally active in specific biological modules and may be potential centers of small community structures [20]. ELC also attains a better EQ performance than the other two methods, with better GO enrichment performance and larger community size as mentioned in the previous section.

The only exception that needs further discussion is the Football network result, where ELC does not reach the best EQ value and the performance appears to be poorer than the other two methods. This unexpected result is notable, since EQ is used by ELC to determine the cutoff on the dendrogram. We think that this could be due to two potential reasons, one is the nodes cover rate, and the other is the network’s structure. Both will be discussed below.

From Table 6 we see that only the ELC method does not prune any node during the computation. The “linkcomm” R package for LC deletes some singular nodes and CPM tends to find the biggest block in the network ignoring smaller ones. After deleting some nodes, the remaining network’s topology structure will appear to be simplified. CPM will produce unions of all k-cliques [5] and LC will generate a denser network [14], consequently the PD and EQ values of LC and CPM will appear to be higher and better than they should be considering also the nodes that are left out. A further improvement direction for ELC could be to weaken the built-in constraint that each node has to belong to a community.

Meanwhile, the structure of the network will significantly influence the final community results, especially for the average degree of nodes and 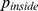. From the artificially-generated datasets results, we can see that when the nodes average degree and 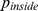 vary over a wide range, the performance of the three methods will vary too. In particular, higher nodes average degree corresponds to more connection opportunities between different communities. When 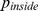 is low, it is hard for hierarchical clustering to individualize the structure and for the EQ measure to divide the tightly integrated network.

Consistently, the Y2H, Karate and Dolphin networks have low average degree, namely around 3, 5 and 5, and corresponding 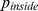 near to 0.9, 0.8 and 0.7, all of which are located in the dominant region of ELC. On the other hand, US politics network has average degree around 9 and 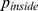 near to 0.7, which is on the overlapping dominant region between ELC and CPM. So this could explain the reason why ELC and CPM have relatively good EQ values on this dataset in Table 5. However, the Football network’s average degree is almost 10 with 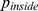 around 0.6, which is just in the lower half of Figure 9. In this region, CPM performs better than ELC and LC, and may be an additional reason why ELC has best EQ performance on all the other four real-world datasets except for the Football network itself.
